# The Portuguese Third Version of the Copenhagen Psychosocial Questionnaire: Preliminary Validation Studies of the Middle Version among Municipal and Healthcare Workers

**DOI:** 10.3390/ijerph19031167

**Published:** 2022-01-21

**Authors:** Teresa P. Cotrim, Pedro Bem-Haja, Anabela Pereira, Cláudia Fernandes, Rui Azevedo, Samuel Antunes, Joaquim S. Pinto, Flávio Kanazawa, Isabel Souto, Elisabeth Brito, Carlos F. Silva

**Affiliations:** 1 Ergonomics Laboratory, Faculdade de Motricidade Humana, Universidade de Lisboa, 1499-002 Cruz-Quebrada, Portugal; kanazawaflavio@uol.com.br; 2 CIAUD, Faculdade de Arquitetura, Universidade de Lisboa, 1349-063 Alto da Ajuda, Portugal; 3 CINTESIS, Department of Education and Psychology, University of Aveiro, 3810-193 Aveiro, Portugal; pedro.bem-haja@ua.pt; 4 CIDTFF, Department of Education and Psychology, University of Aveiro, 3810-193 Aveiro, Portugal; anabelapereira@ua.pt (A.P.); isabel.souto@ua.pt (I.S.); 5 CATIM, Technological Center, 4100-414 Porto, Portugal; claudia.fernandes@catim.pt; 6 Research Unit in Management Sciences and Sustainability (UNICES), University of Maia (UMAIA), 4475-690 Maia, Portugal; razevedo@maieutica.ismai.pt; 7 Center ALGORITMI, University of Minho, 4800-058 Guimarães, Portugal; 8 APPsyCI—Applied Psychology Research Center Capabilities & Inclusion, ISPA—Instituto Universitário, 1149-041 Lisboa, Portugal; ssantunes@ispa.pt; 9 IETA, Departamento de Eletrónica, Telecomunicações e Informática, University of Aveiro, 3810-193 Aveiro, Portugal; jsp@ua.pt; 10 GOVCOPP, School of Technology and Management of Águeda, University of Aveiro, 3810-193 Aveiro, Portugal; ebrito@ua.pt; 11 WJCR, Department of Education and Psychology, University of Aveiro, 3810-193 Aveiro, Portugal; csilva@ua.pt

**Keywords:** COPSOQ III Portuguese middle version, psychosocial risk factors, psychosocial working conditions, preliminary research version, reliability

## Abstract

A third version of the Copenhagen Psychosocial Questionnaire (COPSOQ III) was developed internationally aiming to respond to new trends in working conditions, theoretical concepts, and international experience. This article aims to present the preliminary validation studies for the Portuguese middle version of COPSOQ III. This is an exploratory cross-sectional study viewing the cross-cultural adaption of COPSOQ III to Portugal, ensuring the contents and face validity and performing field-testing in order to reduce the number of items and to obtain insight into the data structure, through classic test theory and item response theory approaches. The qualitative study encompassed 29 participants and the quantitative one 659 participants from municipalities and healthcare settings. Content analysis suggested that minor re-wording could improve the face validity of items, while a reduced version, with 85 items, shows psychometric stability, achieving good internal consistency in all subscales. The COPSOQ III Portuguese middle version proved to be a valid preliminary version for future validation studies with various populations, able to be used in correlational studies with other dimensions.

## 1. Introduction

In a world where psychosocial factors at work have a devastating individual, social and economic impacts, assessing psychosocial factors quickly becomes crucial [1,2,3,4] for individuals, society and economies all over the world. In this sense, assessing and measuring the psychosocial work environment is increasingly seen as essential for the development of healthy workplaces [1,2,3,4]; it has been incorporated in organizational systematic health and safety management systems. This evolution of occupational health and safety systems implied a high demand for reliable and trustful instruments that not only have a research impact but also a practical impact on sustainable business systems.

The Copenhagen Psychosocial Questionnaire (COPSOQ) was developed around the year of 2000 and became a relevant instrument for the assessment of psychosocial factors at work, thus allowing for the improvement the corresponding intervention evaluations [5]. This instrument was revised in 2010, giving place to a new version—the COPSOQ II [6]. Several changes were introduced in this renewed COPSOQ, covering both dimensions and items of the questionnaire [7]. COPSOQ I and II have three versions: short, middle, and long. The short and medium versions were intended to be used in practical contexts, while the long one was meant for research contexts [6,7].

COPSOQ has been translated and validated in several languages [6,7,8,9,10,11]. In Portugal, the validation of COPSOQ II started in 2006 [12], and it has been used ever since in several national studies, for intervention purposes, in a variety of occupations and in workplaces. This past project included the validation of the COPSOQ II middle version [12,13] used in research [14,15,16] and by enterprises in Portugal. The Portuguese middle version comprised nine domains (demands at work, work organization and job contents, interpersonal relations and leadership, work-individual interface, values in the workplace, personality, health and well-being, and offensive behaviors), 29 dimensions (also called scales), and 76 items [12,13]. This is an ongoing collaborative project, started in 2006, that culminated in the creation of the Portuguese Observatory of Psychosocial Factors (Popsy@work) during the year of 2021, engaging universities and technological interface centers.

Meanwhile, the International COPSOQ Network released a revised third version of the questionnaire: COPSOQ III [1,7]. The development of a third version was justified by [7]: Changes in societal trends due to the evolution of work and working conditions related with the increased globalization and computerization, which were somehow intensified during the economic crisis, in 2008; scientific concepts, viewing to a more comprehensive perspective about job demands-resources and job satisfaction, as well as to include the evolution in stress and workplace theories; experience with the use of the questionnaire, which has increased the need for the inclusion of items or word rephrasing to improve the adaptations to different national, cultural, and occupational contexts.

Essentially, this new version will provide one updated instrument allowing comparability between populations and time periods [7,11], responding to the different stakeholders’ needs, and introducing the results of international experiences. More importantly, the third COPSOQ version is designed to allow for a flexible adaptation to national and industry specific contexts without compromising the potential for international comparisons and for comparisons over time [7,11]. This is clearly expressed with the introduction of the core item concept, regarding a group of items that must be present in all versions, but must be supplemented by other items, that can vary between national versions, assuring a new perspective of flexibility that allows for the accommodation of the needs of each country [7]. Short and middle versions of COPSOQ III are still looked as the basis for the use in companies [7].

The COPSOQ middle version provides versatility of uses, allowing an extensive coverage of psychosocial factors allied with the possibility of developing and applying benchmarking practices at several levels—e.g., organization, sectorial or professional occupations, regions, and countries. COPSOQ III has already been adapted and validated in different countries (Canada, Spain, France, Germany, Sweden, Turkey) since 2016 [1,7,17,18]. As a measure of the psychosocial work environment, COPSOQ captures a wide range of psychosocial dimensions [1,7,17,18], which makes it an instrument suitable to meet the needs of psychosocial risks management processes has advocated by ISO 45,003:2021, ISO 45,001 or even ISO 9001, as part of an integrated quality, health and safety management system [19]. Having validated instruments that allow research and practice to move forward is an important asset for organizations that want to add value to their management systems in the scope of continuous improvement and sustainable development.

Following the previous experience with the validation of the COPSOQ II middle version in Portugal, this study aims to present, discuss and evaluate the aspects of cross-cultural adaption, reliability, and preliminary validity of the Portuguese COPSOQ III Middle Version, viewing to obtain a research version.

## 2. Materials and Methods

### 2.1. Study Design

This is an exploratory cross-sectional study aiming the cross-cultural adaption of COPSOQ III to Portugal, by analyzing the face and content validity (qualitatively), and performing field-testing to reduce the number of items and to obtain insight into the data structure, through classic test theory (CTT) and item response theory (IRT) approaches. The main aim of this study is to achieve a research version for future validation studies. It includes two phases: first, a qualitative pilot study, to analyze face validity and to ensure content validity; second, a quantitative field study, to trim the questionnaire through a reliability approach and to confirm the trimmed solution with construct validity and item response theory analysis. These phases are according to the international procedures defined by the International COPSOQ network (https://www.copsoq-network.org/ (accessed on 4 January 2022)), allowing data comparability among different countries and populations.

### 2.2. Participants

For the first phase, 29 participants were invited to participate in the think aloud and gave informed oral consent. The sample included various geographical regions (48.3% from the north, 34.5% from Lisbon and Tagus Valley, 13.8% from the center and 3.4% from the Algarve), 51.7% were women, the mean age was 42.6 years old (±11.3; min = 21; max = 65), with different occupations and education levels (from secondary school (24.1%) to graduation levels (75.9%)).

To trim the version resulting from the thinking aloud procedure (first phase), an on-line version of the questionnaire was sent to a municipality and to healthcare settings. The informed consent was presented in the first page of the questionnaire, which could only be accessed by those who had given their informed consent. The final sample included 659 participants (488 females), aged between 20 and 68 years old, with a mean age of 47.5 years (SD = 9.4). Regarding the professional sectors, 190 participants belonged to the healthcare sector and 469 came from a municipality. This was a convenience sample adequate for this exploratory study.

### 2.3. Instruments

The evolution of the middle version from COPSOQ II to COPSOQ III was mainly based on changes of dimensions and items: the addition of the dimension “control over working time”, the split of the “job insecurity dimension” into two dimensions (“job insecurity” and “insecurity over working conditions”), the addition of items in some dimensions (e.g., the dimension “trust regarding management” was relabeled “vertical trust” and a new item was introduced), the relabeling of some dimensions (e.g., the dimension “rewards” was relabeled “recognition”), and the rephrasing of some items (e.g., items included in the dimension “social support from supervisor” were rephrased to stress that support should be asked when needed) [7]. Based on the knowledge of these changes and on the validation project of the Portuguese COPSOQ II middle version [12], with the corresponding cross-cultural adaptation of the instrument to Portuguese, it was decided to use the tool obtained from the previous validation project (maintaining all the dimensions and the items, but one: “offensive behaviors” will not be included in the middle version of COPSOQ III) and to translate only the new items and scales by using a pool of seven experts from different disciplines (Psychology, Ergonomics, Engineering, and Management), diverse professional backgrounds (academic, industrial), and a consensus technique. The Portuguese COPSOQ II middle version included items and dimensions from the long version [12], and this option was maintained for the COPSOQ III middle version. All items were measured using a 5-points Likert scale [5,6,7,12,13], from “never” until “always” and from “none” until “extremely”. The dimension “Self-rated Health” is measured with a 5-points scale from “Excellent” to “Poor”.

### 2.4. Methods

After participants have completed the questionnaire, the think aloud method was used [20] to ensure the content validity, defined by the COSMIN group as ‘the degree to which the content of an instrument is an adequate reflection of the construct to be measured’ [21], and assess face validity, defined as ‘the degree to which the items of an instrument indeed look as though they are an adequate reflection of the construct measured’ [21]. Participants were asked to complete the questionnaire and, afterwards, to comment on the appropriateness, comprehensibility, relevance, and ambiguity of the items and problems with response categories. Additionally, they were asked on their own interpretation of the different terms [20]. All the comments and the duration to complete the questionnaire were both registered. This step was followed by a content analysis and a qualitative analysis of the results carried out by the pool of seven experts aiming at selecting the relevant changes to be implemented. Considering a participants’ widespread complaint that the questionnaire was too long, thus compromising its completion, the version of the questionnaire that came out of the thinking aloud method was submitted to a trimming procedure. This trimming consisted of three different stages:

1. Trimming based on Reliability Analysis

At this stage, all subscales were submitted to a reliability analysis. Considering the need to reduce the number of items in the instrument, it was decided to carry out an “if item deleted” analysis, eliminating the items that affected reliability in each factor. Considering the ordinal nature of data and the violation of mathematical continuity, internal consistency was assessed through the calculation of Ordinal Cronbach’s α [22], based on a polychoric correlation matrix. Raw alpha (traditionally used based on Pearson’s correlation matrix) and raw omega (traditionally used) values were also provided to allow for comparability between countries, as there are countries that report these indices as a measure of reliability.

2. Agreement on Trimming based on Factorial Validity

The factors that had problematic items in the previous stage were submitted to an exploratory and confirmatory factor analysis to assess whether the elimination of these items would be problematic for the corresponding factor, compromising its factorial validity. Mardia’s test was performed to assess the multivariate normality of the sample [23]. Given the violation of the normality assumption and the ordinal nature of the data, estimators based on the asymptotic covariance matrix were used. These estimators are derived from the polychoric correlation matrix estimated from the observed categorical variables [24]. In order to perform an exploratory factor analysis (EFA), the ULS estimator, based on the diagonal form of the asymptotic covariance matrix, was used [25]. Factor retention criteria was used, as well as a parallel analysis (of the factor analysis) with the ULS method and the Kaiser criteria. A confirmatory factor analysis (CFA) using a weighted least-square-mean and variance adjusted estimator (WLSMV) was also conducted. The overall goodness-of-fit was assessed using the following indexes and cut-off points for “good adjustment”: Chi-square (χ2); comparative fit index (CFI; 0.90 ≤ CFI ≤ 0.95); Tucker–Lewis index (TLI; 0.90 ≤ TLI ≤ 0.95); root mean square error of approximation (RMSEA; 0.05 ≤ RMSEA ≤ 0.70); P[rmsea ≤ 0.05]; and standardized root-mean-residual (SRMR; SRMR < 0.80) [26]. The Average variance extracted (*AVE*) of each factor was calculated using the following formula [27]:

$$\hat{AVE_{j}}=\frac{\sum_{i=1}^{k}\lambda_{ij}^{2}}{\sum_{i=1}^{k}\lambda_{ij}^{2}+\sum_{i=1}^{k}\varepsilon_{ij}}$$

($\lambda_{ij}$ are the standardized factor weights and $\varepsilon_{ij}=1-R_{ij}^{2}\;≅\;1-\lambda_{ij}^{2}$ are the residues of each item). An *AVE* of 0.5 or greater suggests an adequate convergence between the items of each construct [28].

3. Agreement on Trimming Based on the Item Response Theory

In addition to this factorial verification, based on the classical test theory, a polytomous item response theory analysis [29] with a partial credit model was also carried out to support the decision to eliminate items based on reliability. This analysis aims to assess whether any of the items that were eliminated using classical test theory methods are more discriminative of the measured latent trait than the items that were not eliminated. In fact, this analysis was thus based on the discrimination parameter that represents how much the item can differentiate individuals with different latent traits [29]. If the discrimination value of an included item is higher than the excluded one, it means that the individuals with higher and lower ability are more likely to agree in the item that was not excluded. On the other hand, if there is an agreement between methods (ITR and CCT) we have more confidence in the elimination of items.

All of the statistical analyses were performed using *R*.

## 3. Results

This section is divided into the two main phases of the study, content validity (including face validity), and the trimming process that allowed the analysis of reliability and construct validity aiming at the elimination of items and achievement of a research version.

### 3.1. Content Validity

The comments regarding comprehensibility focused on the level of ambiguity and on the difficulties in understanding some questions (Q3 (*n* = 1); Q11 (*n* = 1); Q12 (*n* = 1); Q14 (*n* = 3); Q20 (*n* = 1); Q40 (*n* = 1); Q41 (*n* = 1); Q45 (*n* = 2); Q64 (*n* = 1); Q78 (*n* = 1)), just as the perception of similarity between items (Q11 and Q12 (*n* = 1); Q75 and Q76 (*n* = 1)). Although the items remained, mainly, with a similar phrasal structure, content analysis suggested that minor re-wording could improve the face validity of these items, which resulted in the version used for the next step (Table 1). Regarding the dimension work-life conflict, the concerns with the different mixed organizational models resulting from the pandemic situation, there including working from home have determined the rephrasing of the items, aiming at their neutrality, considering the possibility to have both people, working from home or in the workplaces.

A feasibility analysis recommended an interview, for those with lower education levels, in order to assure the appropriate interpretation of the items.

### 3.2. Trimming Process

The results of the analysis for each dimension/factor as well as the reliability coefficients in each depurative solution are shown in Table 2. Looking at the data in Table 2, we can see that only the dimensions “Possibilities for Development”, “Control over Working Time”, “Vertical Trust”, “Work-Life Conflict” and “Job Satisfaction” have registered improvements in the reliability coefficients with the elimination of items. In fact, using this methodology, 8 items were eliminated: 1 item in “Possibilities for Development”; 2 items in “Control over Working Time”; 1 item in “Vertical Trust”; 2 items in “Work Life Conflict”; and 2 items in “Job Satisfaction”.

After this Trimming stage, and in order to confirm the solution obtained, the trimmed and non-trimmed version of the subscales that were reduced in the previous step were submitted to an exploratory and confirmatory factor analysis. Mardia’s test showed that the data is not multivariate normal, g1p = 1732.29, χSkew = 190263.4, *p* < 0.0001; g2p = 9660.6, ZKurtosis = 79.72, *p* < 0.0001; χSMSkew = 191148, *p* < 0.0001. Bartlett’s test of sphericity (χ^2^ = 42294.85, *p* < 0.001) and KMO (0.934) indicate that this data is probably suitable for factor analysis.

The EFA (factorial weights, variances, and complexity) and CFA (factorial weights, AVE and goodness of fit indexes) results can be seen in Table 3. By analyzing Table 3, it is possible to verify that both the exploratory and confirmatory factorial analysis supported the decisions obtained in the trimming based on reliability. In fact, higher loadings were found and AVEs and better goodness of fit indexes on trimmed subscales than on non-trimmed ones.

The results of the polytomous IRT with the partial credit model can be seen in Table 4.

Remarkably, the items eliminated from each factor present a lower discrimination value in this analysis. This result showed a congruence between analytical methods (CTT vs. IRT) and gives us confidence that the elimination of the eight items does not affect the reliability and validity of the factors and the instrument.

## 4. Discussion

Worldwide, more than one in four older workers experience job strains related to work organization and the absence of an adequate assessment of psychosocial risk factors [30]. The relevance of assessing and monitoring psychosocial risk factors in workplaces is clear. It means that companies need accurate and updated information to support their decisions, as well as usable tools to assess the psychosocial factors. COPSOQ II has played an important role in the assessment of psychosocial factors all over the world [5,6,8,9], especially in Portugal [12,13,19], with a wide use in academic studies [14,15] and in business environments [19]. With the emergence of COPSOQ III, which includes fundamental dimensions for the psychosocial reality at work, it became essential to validate the Portuguese middle version.

In a first phase, we intended to evaluate the face validity and to ensure the content validity of the new version of this widely used instrument. Meeting and reflecting with experts, along with the thinking aloud procedure with study subjects, led to changes in the formulation of some items, in order to make them fit the Portuguese culture and working contexts and to ensure the appropriate and accurate interpretation of the items by every subject, regardless of the academic background, work experience, gender or age. This step allowed the instrument to be globally understood, becoming more inclusive and gender- and age-neutral. Moreover, the changes that emerged from the COVID-19 pandemic, regarding mixed organizational models that include working from home [31], were considered in order to obtain a version that could be used in different scenarios. This first phase ended with a version that comprised 31 dimensions and 93 items. The Portuguese COPSOQ III middle version includes items from the long version to the middle version. This is a common procedure accepted by the international COPSOQ network [7] and used worldwide in validation studies of the third version [17].

Considering that one of the most frequent complaints recorded during the thinking aloud process was the length of the questionnaire, which made the process of completing it very time consuming, or even leading to dropouts, a trimming procedure was carried out. This procedure was based on the psychometrics of the classical test theory and the TRI showed the psychometric robustness of the trimmed version. The Portuguese COPSOQ III middle version started with 24 new items, when compared with the Portuguese COPSOQ II middle version. In the trimmed version, eight items were excluded. Mostly, they were part of already existing dimensions (development possibilities, vertical trust, work-life conflict, and job satisfaction) and one new one (control over working time). Interestingly, other validation studies faced similar problems with internal consistency, with almost the same dimensions: development possibilities, work-life conflict, job satisfaction, and control over working time [17]. The international validation studies have already recommended that the selection of items within the scales could be reconsidered, aiming to overcome problems with internal consistency or psychometric robustness [7]. In accordance with other validation studies that used a working population for different activity sectors [17], or those focused on specific populations, such as professional drivers [18], improvements in the reliability coefficients with the elimination of items were obtained.

It is important to stress that, in the present study, the internal consistency was even stronger than the mean values presented in the original evaluation study [7]. These results show that there might be slightly different versions of COPSOQ III depending on the process of adaptation and validation at a national level, or towards specific populations [7,17,18], which, however, always allow data comparability due to the followed common and validated methodological approach. Nevertheless, in this Portuguese middle version, none of the eliminated items was “core items”, thus respecting the concept introduced in the development of COPSOQ III. Core items are considered mandatory to ensure comparability between countries. However, at the same time, national versions can diverge regarding supplementary items [7]. Despite the procedure described, the Portuguese COPSOQ III middle version maintained its coherence and alignment with the original international instrument [7] and its several validations worldwide [1,7,17,18].

This preliminary validation study was carried out with a convenience sample that consisted mainly of the healthcare sector and of municipalities; the response rate could not be calculated. The data was collected in early 2021, a year that was strongly affected by the worldwide COVID-19 pandemic, the volatile country dynamics, and the ever-changing working environments and work regulations. This fact is prone to have an impact on perceptions or even on responses; however, it is a common scenario shared by every country in the world [32].

It should also be noted that the objective was to adapt and to perform a preliminary assessment of the validity of the scales; therefore, important procedures, such as criterion validity (external criteria), factorial validity with global models, scalar configurational metric invariance analysis between professional sectors, and convergent and discriminant validity, were not assessed in this study. Taking future research into consideration, additional studies would be needed in order to analyze the overall structure of the Portuguese COPSOQ III middle version with various populations, based on construct validity, external validity, and predictive validity.

In addition to the need for a rigorous analysis of the construct and criterion validities, there is a need, in future steps of the validation process, to expand the sample to other occupational settings in order to obtain a comprehensive analysis, all the while not compromising the data validity, either for research contexts or working environment assessments and interventions. Despite that, the sample size served the objectives of the study, methodology and study phase.

This Portuguese middle version of COPSOQ III proved to be a valid preliminary version for future validation studies. Although this initial part of research is not reported frequently in the literature, it was considered of importance to show how the research version was achieved regarding transparency in research and data transferability to organizational settings.

## 5. Conclusions

The Portuguese research version of the COPSOQ III Middle Version presented good face validity, ensured content validity, and reached a stable reduced version of 85 items while maintaining good psychometric characteristics. The Portuguese version proved to be a valid preliminary version for future validation studies with various populations, and for use in correlational studies with other dimensions. The instrument also has a high potential for transferring knowledge from academia to industrial and/or occupational scenarios.

It should also be noted that the COPSOQ III Middle version is a useful tool to assess the psychosocial risk factors and to contribute to health and well-being at work, which is considered a universal value by the United Nations (UN), the World Health Organization (WHO) and the International Labour Organization (ILO). This version also has the advantage of contributing to studies in the area of occupational health and safety, thus playing an important role in sustainable development and contributing to achieving the goals of the 2030 Agenda for Sustainable Development.

## Figures and Tables

**Table 1:** Domains, Dimensions and Items of the Portuguese Middle Version of COPSOQ III in Portuguese/English with the identification of new items and dimensions (identified in italics in the text).

Domains	Dimensions	Items
Exigências Laborais/Demands at Work	Exigências Quantitativas/Quantitative Demands	Q1. A sua carga de trabalho acumula-se por ser mal distribuída?/Is your workload unevenly distributed so it piles up?
		*Q2. Com que frequência fica com trabalho atrasado?/Do you get behind with your work? (New)*
		Q3. Com que frequência não tem tempo para completar todas as tarefas do seu trabalho?/How often do you not have time to complete all your work tasks?
	Ritmo de Trabalho/Work Pace	Q4. Precisa de trabalhar muito rapidamente? Do you have to work very fast?
		*Q5. Trabalha a um ritmo elevado ao longo de toda a jornada de trabalho?/Do you work at a high pace throughout the day? (New)*
	Exigências Cognitivas/Cognitive Demands	Q6. O seu trabalho exige a sua atenção constante?/Do you have to keep your eyes on lots of things while you work?
		*Q7. O seu trabalho requer que memorize muitas informações?/Does your work require that you remember a lot of things? (New)*
		Q8. O seu trabalho requer que seja bom a propor novas ideias?/Does your work demand that you are good at coming up with new ideas?
		Q9. O seu trabalho exige que tome decisões difíceis?/Does your work require you to make difficult decisions?
	Exigências Emocionais/Emotional Demands	*Q10. O seu trabalho coloca-o/a em situações emocionalmente perturbadoras?/Does your work put you in emotionally disturbing situations? (New)*
		*Q11. No seu trabalho tem de lidar com os problemas pessoais de outras pessoas?/Do you have to deal with other people’s personal problems at work? (New)*
		Q12. O seu trabalho exige emocionalmente de si? Is your work emotionally demanding?
Organização do Trabalho e Conteúdo/Work Organization and Job Contents	Influência no Trabalho/Influence at Work	Q13. Tem um elevado grau de influência nas decisões sobre o seu trabalho?/Do you have a large degree of influence on the decisions concerning your work?
		Q14. Pode influenciar a quantidade de trabalho que lhe compete a si?/Can you influence the amount of work assigned to you?
		Q15. Tem alguma influência sobre o tipo de tarefas que faz?/Do you have any influence on what you do at work?
		*Q16. Tem alguma influência sobre o modo como faz o seu trabalho?/Do you have any influence on how you do your work? (New)*
	Possibilidades de Desenvolvimento/Development Possibilities	Q17. O seu trabalho permite-lhe aprender coisas novas?/Do you have the possibility of learning new things through your work?
		Q18. O seu trabalho permite-lhe usar as suas competências ou capacidades? Can you use your skills or expertise in your work?
		*Q19. O seu trabalho permite-lhe desenvolver as suas competências?/Does your work give you the opportunity to develop your skills? (New)*
		Q20. O seu trabalho requer que tenha iniciativa? Does your work require initiative?
	Controlo sobre o tempo de Trabalho/Control over Working Time *(New)*	*Q21. Pode decidir quando faz as suas pausas? Can you decide when to take a break?*
		*Q22. Pode tirar férias mais ou menos quando deseja?/Can you take holidays more or less when you wish?*
		*Q23. Pode fazer uma pausa no trabalho para falar com um/a colega?/Can you leave the work to have a chat with a colleague?*
		*Q24. Se tiver um assunto pessoal para tratar, consegue deixar a sua tarefa por meia hora sem precisar de autorização?/If you have some private business is it possible for you to leave your piece of work for half an hour without special permission?*
		*Q25. Precisa de fazer horas extra?/Do you have to do overtime? (moved from the dimension quantitative demands from COPSOQ II)*
	Significado do Trabalho/Meaning of Work	Q26. O seu trabalho tem algum significado para si?/Is your work meaningful?
		Q27. Sente que o seu trabalho é importante?/Do you feel that the work you do is importante?
		Q28. Sente-se motivado e envolvido com o seu trabalho?/do you feel motivated and involved in your work?
Interface Trabalho—Indivíduo/Work Individual Interface	Compromisso face ao Local de Trabalho/Commitment to the Workplace	Q29. Gosta de falar com os outros sobre o seu local de trabalho?/Do you enjoy telling others about your place of work?
		Q30. Sente que os problemas do seu local de trabalho são seus também?/Do you feel that the problems at your place of work are yours also?
		*Q 31. Sente orgulho em pertencer a esta organização/empresa? Are you proud of being part of this organization? (New)*
Relações Sociais e Liderança/Interpersonal Relations and Leadership	Previsibilidade/Predictability	Q32. No seu local de trabalho é informado/a com antecedência sobre decisões importantes, mudanças ou planos para o futuro?
		Q33. Recebe toda a informação de que necessita para fazer bem o seu trabalho?
	Reconhecimento/Recognition	Q34. O seu trabalho é reconhecido e apreciado pela gestão de topo?/Is your work recognized and appreciated by the management?
		Q35. A gestão de topo do seu local de trabalho respeita-o/a?/Does the management at your workplace respect you?
		Q36. É tratado/a de forma justa no seu local de trabalho?/Are you treated fairly at your workplace?
	Transparência do Papel Laboral/Role Clarity	Q37. O seu trabalho tem objectivos claros?/Does your work have clear objectives?
		Q38. Sabe exatamente quais as suas responsabilidades?/Do you know exactly which areas are your responsability?
		Q39. Sabe exatamente o que é esperado de si?/Do you know exactly what is expected of you at work?
	Conflito de Papéis Laborais/Role Conflicts	Q40. Faz coisas no seu trabalho com que uns concordam mas outros não?/Are contradictory demands placed on you at work?
		Q41. Por vezes tem que fazer coisas que deveriam ser feitas de outra maneira?/Do you sometimes have to do things wich ought to have been done in a diferente way?
		Q42. Por vezes tem que fazer coisas que considera desnecessárias?/Do you sometimes have to do things wich seem to be unnecessary?
	Qualidade da Liderança/Quality of Leadership	Em relação à sua chefia directa, até que ponto considera que…/To what extent would you say that your immediate superior…
		Q43. Oferece aos indivíduos e ao grupo boas oportunidades de desenvolvimento/formação? Makes sure that the members of staff has good development opportunities?
		Q44. Faz um bom planeamento do trabalho?/is good at work planning?
		Q45. É eficaz a resolver conflitos?/is good at solving conflicts?
		Q46. Dá prioridade à satisfação no trabalho?/gives high priority to job satisfaction?
	Suporte Social de Colegas/Social Support from Colleagues	Q47. Com que frequência tem ajuda e apoio dos seus colegas de trabalho, se necessário?/How often do you get help and support from your colleagues, if necessary?
		Q48. Com que frequência os seus colegas estão recetivos a ouvi-lo/a sobre os seus problemas de trabalho, se necessário?/How often are your colleagues willing to listen to your problems, if needed?
		Q49. Com que frequência os seus colegas falam consigo sobre o seu próprio desempenho laboral?/How often do your colleagues talk with you about how well you carry your own work?
	Suporte Social de Superiores/Social Support from Supervisors	Q50. Com que frequência a sua chefia direta fala consigo sobre como está a decorrer o seu trabalho?/How often is your immediate superior willing to listen to your problems at work, if needed?
		Q51. Com que frequência tem ajuda e apoio da sua chefia direta, se necessário?/How often do you get help and support from your immediate superior, if needed?
		Q52. Com que frequência a sua chefia direta fala consigo sobre o seu desempenho laboral?/How often does your immediate superior talk with you about how well you carry out your work?
	Sentido de Pertença a Comunidade/Sense of Community at Work	Q53. Existe um bom ambiente de trabalho entre si e os seus colegas?/Is there a good atmosphere between you and your colleagues?
		Q54. No seu local de trabalho sente-se parte de uma comunidade?/Do you feel part of a community at your place of work?
		Q55. Existe uma boa cooperação entre os colegas de trabalho?/Is there good co-operation between the colleagues at work?
Interface Trabalho—Indivíduo/Work Individual Interface	Insegurança Laboral/Job Insecurity	Q56. Sente-se preocupado/a em ficar desempregado/a?/Are you worried about becoming unemployed?
		*Q57. Sente-se preocupado/a com a dificuldade em encontrar outro trabalho se ficar desempregado/a?/Are you worried about it being difficult for you to find another job if you became unemployed? (New)*
	Insegurança com as Condições de Trabalho/Insecurity over Working Conditions *(New)*	*Q58. Sente-se preocupado/a em ser transferido/a para outro posto de trabalho contra a sua vontade?/Are you worried about being transferred to another job against your will?*
		*Q59. Preocupa-o/a que o seu horário de trabalho (turno, dias úteis, hora de entrada e saída…) seja mudado contra sua vontade?/Are you worried about the timetable being changed (shift, weekdays, time to enter and leave, …) against your will?*
		*Q60. Sente-se preocupado/a com uma diminuição na sua retribuição (redução, introdução de remuneração variável…)?/Are you worried about a decrease in your salary (reduction, variable pay being introduced…)?*
	Qualidade do trabalho/Quality of Work *(New)*	*Q61. Está satisfeito (a) com a qualidade do trabalho realizado?/Are satisfied with the quality of the work performed at your workplace?*
Capital Social/Social Capital	Confiança Horizontal/Horizontal Trust	Q62. Os trabalhadores confiam uns nos outros de um modo geral?/Do the employees in general trust each other?
		Q63. Os trabalhadores ocultam informações uns dos outros?/Do the employees withhold information from each other?
		Q64. Os trabalhadores ocultam informação à gestão de topo?/Do the employees withhold information from the management?
	Confiança Vertical/Vertical Trust	Q65. A gestão de topo confia nos seus trabalhadores para fazerem o seu trabalho bem?/Does the management trust the employees to do their work well?
		Q66. Os trabalhadores confiam na informação que lhes é transmitida pela gestão de topo?/Can the employees trust the information that comes from the management?
		*Q67. Os trabalhadores podem expressar as suas opiniões à gestão de topo?/Are the employees able to express their views and feelings? (New)*
		Q68. A gestão de topo oculta informação aos seus trabalhadores?/Does the management withhold important information from the employees?
	Justiça Organizacional/Organizational Justice	Q69. Os conflitos são resolvidos de uma forma justa?/Are conflicts resolved in a fair way?
		Q70. O trabalho é distribuído de forma justa?/Is the work distributed fairly?
		Q71. As sugestões dos trabalhadores são tratadas de forma séria pela gestão de topo?/Are all suggestions from employees treated seriously by the management?
		*Q72. Quando os trabalhadores fazem um bom trabalho são reconhecidos?/Are employees appreciated when they have done a good job? (New)*
Interface Trabalho—Indivíduo/Work Individual Interface	Conflito Trabalho-Família/Work-Life Conflict	*Q73. Há momentos em que precisa de estar a trabalhar e gerir a vida privada/familiar ao mesmo tempo?/Are there times when you need to be at work and at home at the same time? (New)*
		Q74. Sente que o seu trabalho lhe exige tanta energia, que acaba por afetar a sua vida privada/familiar negativamente?/Do you feel that your work drains so much of your energy that it has a negative effect on your private life?
		Q75. Sente que o seu trabalho lhe exige tanto tempo, que acaba por afetar a sua vida privada/familiar negativamente?/Do you feel that your work takes so much of your time that it has a negative effect on your private life?
		*Q76. As exigências do seu trabalho interferem com a sua vida privada e familiar?/The demands of my work interfere with my private and family life (New)*
		*Q77. Devido ao seu trabalho tem que alterar os seus planos familiares e pessoais?/Due to work-related duties, I have to make changes to my plans for private and family activities? (New)*
	Satisfação com o Trabalho/Job Satisfaction	Em relação ao seu trabalho em geral, quão satisfeito está com…/Regarding your work in general. How pleased are you with…
		Q78. As suas perspetivas de trabalho?/your work prospects?
		Q79. O seu trabalho de uma forma global?/your job as a whole, everything taken into consideration?
		*Q80. O seu salário/retribuição?/your salary? (New)*
		Q81. As condições físicas do seu local de trabalho?/the physical working conditions?
		Q82. A forma como as suas capacidades e competências são usadas?/the way your abilities are used?
Saúde e Bem-Estar/Health and Well-Being	Auto-Avaliação da Saúde/Self-Rated Health	Q83. Em geral, sente que a sua saúde é:/In general, would you say your health is:
Personalidade/ Personality	Auto-Eficácia/Self-Efficacy	Q84. Sou sempre capaz de resolver problemas se tentar o suficiente./I am always able to solve difficult problems, if I try hard enough.
		Q85. É fácil seguir os meus planos e atingir os meus objectivos./I tis easy for me to stick to my plans and reach my objectives.
Saúde e Bem-Estar/Health and Well-Being	Problemas de Sono/Sleeping Troubles	Q86. Sentiu dificuldade em adormecer?/How often have you found it hard to go to sleep?
		Q87. Acordou várias vezes durante a noite e depois não conseguia adormecer novamente?/How often have you woken up several times and found it difficult to get back to sleep?
	Burnout	Q88. Tem-se sentido fisicamente exausto/a?/How often have you been physically exhausted?
		Q89. Tem-se sentido emocionalmente exausto/a?/How often have you been emotionally exhausted?
	Stress	Q90. Tem-se sentido irritado/a?/How often have you been irritable?
		Q91. Tem-se sentido ansioso/a?/How often have you been tense?
	Sintomas Depressivos/Depressive Symptoms	Q92. Tem-se sentido triste?/How often have you felt sad?
		Q93. Tem sentido falta de interesse por coisas do quotidiano?/How often have you lacked interest in everyday things?

**Table 2:** Reliability analysis in pre and post trimming solutions.

	PRE—TRIMMING					POST—TRIMMING				
DIMENSION/FACTOR	Ni ^1^	AVG Poly	Ord. α	Raw α	Raw ω	Ni	AVG Poly	Ord. α	Raw α	Raw ω
Quantitative Demands	3	0.88	0.88	0.84	0.85	3	Same			
Work Pace	2	-	-	0.84	0.84	2	Same			
Cognitive Demands	4	0.42	0.75	0.69	0.70	4	Same			
Emotional Demands	3	0.77	0.91	0.88	0.88	3	Same			
Influence at Work	4	0.56	0.84	0.81	0.82	4	Same			
Development Possibilities	4	0.68	0.89	0.85	0.86	3 ^2^	0.76	0.90	0.86	0.88
Control over Working time	5	0.23	0.60	0.61	0.67	3 ^3^	0.52	0.76	0.72	0.72
Meaning of Work	3	0.74	0.90	0.85	0.86	3	Same			
Commitment to the Workplace	3	0.42	0.69	0.64	0.65	3	Same			
Predictability	2	-	-	0.77	0.77	2	Same			
Recognition	3	0.76	0.91	0.87	0.88	3	Same			
Role Clarity	3	0.73	0.88	0.84	0.85	3	Same			
Role Conflicts	3	0.58	0.81	0.76	0.78	3	Same			
Quality of Leadership	4	0.79	0.94	0.92	0.92	4	Same			
Social Support from Colleagues	3	0.62	0.83	0.78	0.80	3	Same			
Social Support from Supervisor	3	0.77	0.91	0.88	0.89	3	Same			
Sense of Community at Work	3	0.82	0.93	0.89	0.89	3	Same			
Job Insecurity	2	-	-	0.86	0.86	2	Same			
Insecurity over Working Conditions	3	0.57	0.80	0.75	0.76	3	Same			
Quality of Work	1					1				
Horizontal Trust	3	0.60	0.82	0.77	0.79	3	Same			
Vertical Trust	4	0.57	0.84	0.79	0.82	3 ^4^	0.72	0.88	0.84	0.85
Organizational Justice	4	0.70	0.90	0.87	0.87	4	Same			
Work Life Conflict	5	0.76	0.94	0.92	0.93	3 ^5^	0.91	0.97	0.95	0.95
Job Satisfaction	5	0.55	0.85	0.82	0.84	3 ^6^	0.75	0.90	0.86	0.87
Self-Rated Health	1					1				
Self-Efficacy	2	-	-	0.69	0.69	2	Same			
Sleeping Troubles	2	-	-	0.85	0.85	2	Same			
Burnout	2	-	-	0.88	0.88	2	Same			
Stress	2	-	-	0.86	0.87	2	Same			
Depressive Symptoms	2	-	-	0.86	0.86	2	Same			
Total number of items	93					85				

Ni ^1^ = Number of Items; ^2^ Without item Q20; ^3^ Without item Q24 and item Q25; ^4^ Without item Q68; ^5^ Without item Q73 and item Q77; ^6^ Without item Q80 and item Q81.

**Table 3:** Exploratory and confirmatory factor analysis for each of the factors to be reduced.

	EFA			CFA		
	Loading (λ)	r^2^	u^2^	Loading (λ)	AVE	Goodness and Badness of Fit
PD Full Item 17	0.71	0.51	0.49	0.71	0.61	CFI = 0.99 TLI = 0.98 RMSEA = 0.02 SRMR = 0.01
Item 18	0.87	0.76	0.24	0.87		
Item 19	0.88	0.78	0.22	0.88		
Item 20	0.63	0.40	0.60	0.64		
PD Trimmed Item 17	0.70	0.49	0.51	0.70	0.68	CFI = 1 SRMR = 0 TLI = 1 RMSEA *p* < 0.001
Item 18	0.88	0.77	0.23	0.88		
Item 19	0.89	0.79	0.21	0.89		
COWT Full Item 21	0.74	0.55	0.45	0.74	0.30	CFI = 0.85 TLI = 0.71 RMSEA = 0.131 SRMR = 0.06
Item 22	0.57	0.33	0.67	0.57		
Item 23	0.74	0.55	0.45	0.75		
Item 24	0.46	0.21	0.79	0.46		
Item 25	−0.13	0.02	0.98	−0.12		
COWT Trimmed Item 21	0.73	0.53	0.47	0.73	0.48	CFI = 1 SRMR = 0 TLI = 1 RMSEA *p* < 0.001
Item 22	0.59	0.35	0.65	0.59		
Item 23	0.72	0.52	0.48	0.72		
VT Full Item 65	0.79	0.63	0.37	0.79	0.52	CFI = 1 TLI = 0.99 RMSEA = 0.03 SRMR = 0.01
Item 66	0.85	0.72	0.28	0.85		
Item 67	0.77	0.59	0.41	0.77		
Item 68	−0.46	0.21	0.79	−0.46		
VT Trimmed Item 65	0.81	0.65	0.35	0.81	0.64	CFI = 1 SRMR = 0 TLI = 1 RMSEA *p* < 0.001
Item 66	0.85	0.72	0.28	0.85		
Item 67	0.75	0.57	0.43	0.75		
WLC Full Item 73	0.62	0.38	0.62	0.62	0.74	CFI = 0.99 TLI = 0.98 RMSEA = 0.06 SRMR = 0.02
Item 74	0.90	0.81	0.19	0.90		
Item 75	0.94	0.89	0.11	0.94		
Item 76	0.93	0.87	0.13	0.93		
Item 77	0.82	0.67	0.33	0.82		
WLC Trimmed Item 74	0.91	0.82	0.18	0.91	0.86	CFI = 1 SRMR = 0 TLI = 1 RMSEA *p* < 0.001
Item 75	0.96	0.91	0.09	0.96		
Item 76	0.92	0.85	0.15	0.92		
JS Full Item 78	0.87	0.76	0.24	0.86	0.49	CFI = 0.92 TLI = 0.83 RMSEA = 0.12 SRMR = 0.05
Item 79	0.80	0.65	0.35	0.79		
Item 80	0.53	0.28	0.72	0.53		
Item 81	0.58	0.28	0.72	0.53		
Item 82	0.78	0.61	0.39	0.80		
JS Trimmed Item 78	0.96	0.92	0.08	0.91	0.69	CFI = 1 SRMR = 0 TLI = 1 RMSEA *p* < 0.001
Item 79	0.85	0.66	0.34	0.55		
Item 82	0.72	0.22	0.78	0.72		

PD—Possibilities for Development; COWT—Control over Working time; VT—Vertical Trust; WLC—Work Life Conflict; JS—Job Satisfaction.

**Table 4:** Polytomous IRT analysis with parameter discrimination for each of the target factors.

Subscale/Factor	Itens	Discrimination
Development Possibilities	Item.17	2.064
	Item.18	4.138
	Item.19	4.158
	**Item.20**	**1.709**
Control over Working time	Item.21	2.291
	Item.22	1.475
	Item.23	2.208
	**Item.24**	**1.035**
	**Item.25**	**−0.266**
Vertical Trust	Item.65	3.11
	Item.66	3.964
	Item.67	2.436
	**Item.68**	**−1.281**
Work Life Conflict	**Item.73**	**1.431**
	Item.74	2.824
	Item.75	3.737
	Item.76	3.498
	**Item.77**	**2.587**
Job Satisfaction	Item.78	3.763
	Item.79	3.501
	**Item.80**	**1.128**
	**Item.81**	**1.117**
	Item.82	2.257

In bold are the items that present a lower discrimination value.

## Data Availability

The data presented in this study are available on request from the corresponding author. The data are not publicly available due to privacy and ethical restrictions.
